# Therapeutics of Infancy and Childhood

**Published:** 1899-03

**Authors:** 


**Therapeutics of Infancy and Childhood.** By A. JACOBI, M.D. Second Edition. Pp. xvi., 9—629. Philadelphia: J. B. Lippincott Company. 1898.

It is a pleasure to find that some medical man can avoid the stiff and stilted style that is commonly adopted when giving opinions. Dr. Jacobi writes easily—almost conversationally, and frequently forcibly—so that the task of reading a big book like this is comparatively slight. Unless this is a natural faculty an author may, to create a sensation, overstep the bounds of easy writing and become ridiculous and abusive. We regret to say that Dr. Jacobi has sometimes done this; and we think that the author's disapproval of Emil Behring's methods might have been expressed in less severe language with advantage.

Apart from these blemishes, the book is a very useful one, and contains the opinions and experiences of an acute observer, and as such will be of great value to students of children's diseases. There are things which we do not agree with, but that possibly is inevitable. For instance, in this country Rötheln is looked upon as a separate disease, and though acute rheumatism is common among children it is not in our experience frequently seen in infants. Scrofula we generally take to be synonymous with tubercle, but Dr. Jacobi thinks that the bacillus need not be present; and we must express our surprise at ossification of the costal cartilages (and consequent want of expansion of the lung) being a cause of tubercular disease of the lungs.

